# Simultaneous robotic-assisted prostatectomy and rectal resection: a systematic review

**DOI:** 10.1007/s11701-025-02395-1

**Published:** 2025-05-23

**Authors:** Harry Collin, Benjamin Huang, Amila Siriwardana, Craig Harris, Andrew Stevenson, Anojan Navaratnam, Rachel Esler, Matthew J. Roberts

**Affiliations:** 1 Faculty of Medicine, The University of Queensland, Brisbane, QLD Australia; 2 Department of Urology, Royal Brisbane & Women’s Hospital, Brisbane, QLD Australia; 3 Department of Colorectal Surgery, Royal Brisbane & Women’s Hospital, Brisbane, QLD Australia; 4 University of Queensland Centre for Clinical Research, Brisbane, QLD Australia

**Keywords:** Prostate cancer, Rectal cancer, Robotic surgery, Prostatectomy, Abdominoperineal resection (APR), Anterior resection (AR)

## Abstract

Prostate cancer (PC) and rectal cancer (RC) are common and can require complex management, especially when presenting synchronously or with invasive characteristics. Robotic surgery has emerged as a viable option for managing these challenging cases; however, the outcomes are yet to be summarised or compared to traditional approaches. This systematic review aims to evaluate the feasibility, oncological outcomes, and functional results of simultaneous robotic-assisted resections of the prostate and rectum. Following prior protocol registration (PROSPERO: CRD42023449872) and according to the Preferred Reporting Items for Systematic Reviews and Meta-analysis (PRISMA) guidelines, we conducted a systematic search across multiple databases, selecting studies that involved simultaneous bladder-sparing robotic-assisted prostatectomy and rectal resection. The Joanna Briggs Institute tool was used for risk of bias assessment. Data on patient demographics, treatment details, post-operative course, and functional and oncological outcomes were extracted and analysed. From 1357 identified records, 25 studies met our inclusion criteria resulting in 45 cases/patients. The median patient age was 62 years. Diverse treatment backgrounds and outcome definitions were recorded. Median operative time was 548 min and median estimated blood loss was 450 mL. The overall complication rate was 44.4%, with major complications (Clavien–Dindo III) occurring in 17.8% of cases. Surgical margins were negative in 92.6% of cases. Continence recovery occurred for most (78.6%) of the cases reporting on urinary function. Simultaneous robotic-assisted prostatectomy and rectal resection appears to be a feasible, safe and oncologically effective approach with satisfactory oncological and functional outcomes for managing patients with synchronous or invasive PC and RC.

**Supplementary Information:**

The online version contains supplementary material available at 10.1007/s11701-025-02395-1.

## Introduction

Prostate cancer (PC) and colorectal cancer are the second and third most commonly diagnosed cancer in men [1, 2]. Consequently, dual malignancy is not uncommon [3], while rates of synchronous detection of PC and rectal cancer (RC) may be increasing due to advancements in cancer staging, particularly use of multiparametric MRI (mpMRI) and positron emission tomography (PET) imaging [4]. Up to 10% of RC invade adjacent organs, such as the prostate, which can warrant multi-organ resection [5], while rectal invasion from PC occurs but is less common [6, 7].

Pelvic exenteration has been historically preferred for multi-organ invasive pelvic malignancy, but is technically complex with associated morbidity and mortality [8]. A prostatectomy-only approach may enable bladder preservation to avoid morbidity of cystectomy with urinary diversion and may improve functional outcomes. Open bladder-sparing resection is oncologically feasible for invasive RC [9, 10] and synchronous malignancy [11]. Comparison with cystoprostatectomy showed no compromise in surgical margin rates and survival, with acceptable urinary function [12], but remains uncommon and technically challenging. A pervading concern with bladder preservation is the integrity and longevity of vesicourethral anastomosis, commonly within an irradiated pelvis due to contemporary multimodal treatment approaches.

Growth in use of robotic surgery has spread into exenteration procedures, which are feasible with potentially improved oncological outcomes due to better visualisation and dexterity [13]. Robotic-assisted laparoscopic prostatectomy (RALP) is among the most common uses for a robotic approach [14], while use for rectal resections is growing [15]. Robotic surgery has consistently demonstrated perioperative benefits (lower blood loss and length of stay) compared to open surgery [14, 16], which may be extended towards more complex combined resections with urinary/bowel anastomosis due to known reductions in leak rates with robotic techniques [17] and increased morbidity risk when performing a RALP with previous pelvic surgery [18]. Currently, no summary on simultaneous RALP and rectal resection exists. The aim of this systematic review was to collate and review available data to assess feasibility, oncological and functional outcomes of simultaneous robotic resections of the prostate and rectum.

## Methods

### Study design

A systematic review was conducted based on guidelines published by the Cochrane Collaboration and reported in accordance with the Preferred Reporting Items for Systematic Reviews and Meta-analysis (PRISMA) guidelines [19]. The study protocol was registered with PROSPERO (CRD42023449872).

The literature search was performed in PubMed, Embase, Web of Science and Cochrane Library up to August 2024. Terms encompassing prostate cancer, rectal cancer and robotic surgery were combined. The following terms were combined to capture relevant publications: rectal cancer, rectal neoplasm, colorectal surgery, anterior resection, prostatectomy, prostate adenocarcinoma, prostate neoplasm, prostate cancer, robotic surgical procedures, robotic surgery, and robot-assisted surgery. Reference lists of relevant results were screened for additional studies.

### Selection criteria

Articles were assessed for eligibility for the systematic review using PICOS criteria [20]. Articles were included if participants had undergone simultaneous robotic-assisted prostatectomy and rectal resection. Articles were excluded if other organs or bowel discontinuous with the rectum were resected. All results were considered including case series, case reports, video vignettes and conference abstracts. There were no publication date or language restrictions.

Two authors reviewed the results independently to select relevant articles. Discrepancies were resolved upon discussion between the two reviewers. Risk of bias assessment was performed independently by the two reviewers using the Joanna Briggs Institute tool for case reports and case series [21], as recommended by the Cochrane Collaboration.

### Data extraction

Data extraction was performed independently by two reviewers. Extracted data included patient demographics, co-morbidities, neo-adjuvant treatment, procedure duration, estimated blood loss, intra-operative complications, admission duration, indwelling urinary catheter (IDC) duration, post-operative complications, urinary continence, bowel continence, erectile function, surgical margin status, cancer recurrence, adjuvant treatment and other therapies. Discrepancies were resolved by discussion between the two reviewers.

Patient characteristics and secondary outcomes were summarised using frequency and percent for categorical variables, mean and standard deviations for normally distributed continuous variables and median and interquartile range for non-normally distributed continuous variables. Surgical complications were categorised using the Clavien–Dindo classification [22]. These were further categorised into minor (Grade I and II) and major (Grade III and IV) complications.

## Results

**Fig. 1:**
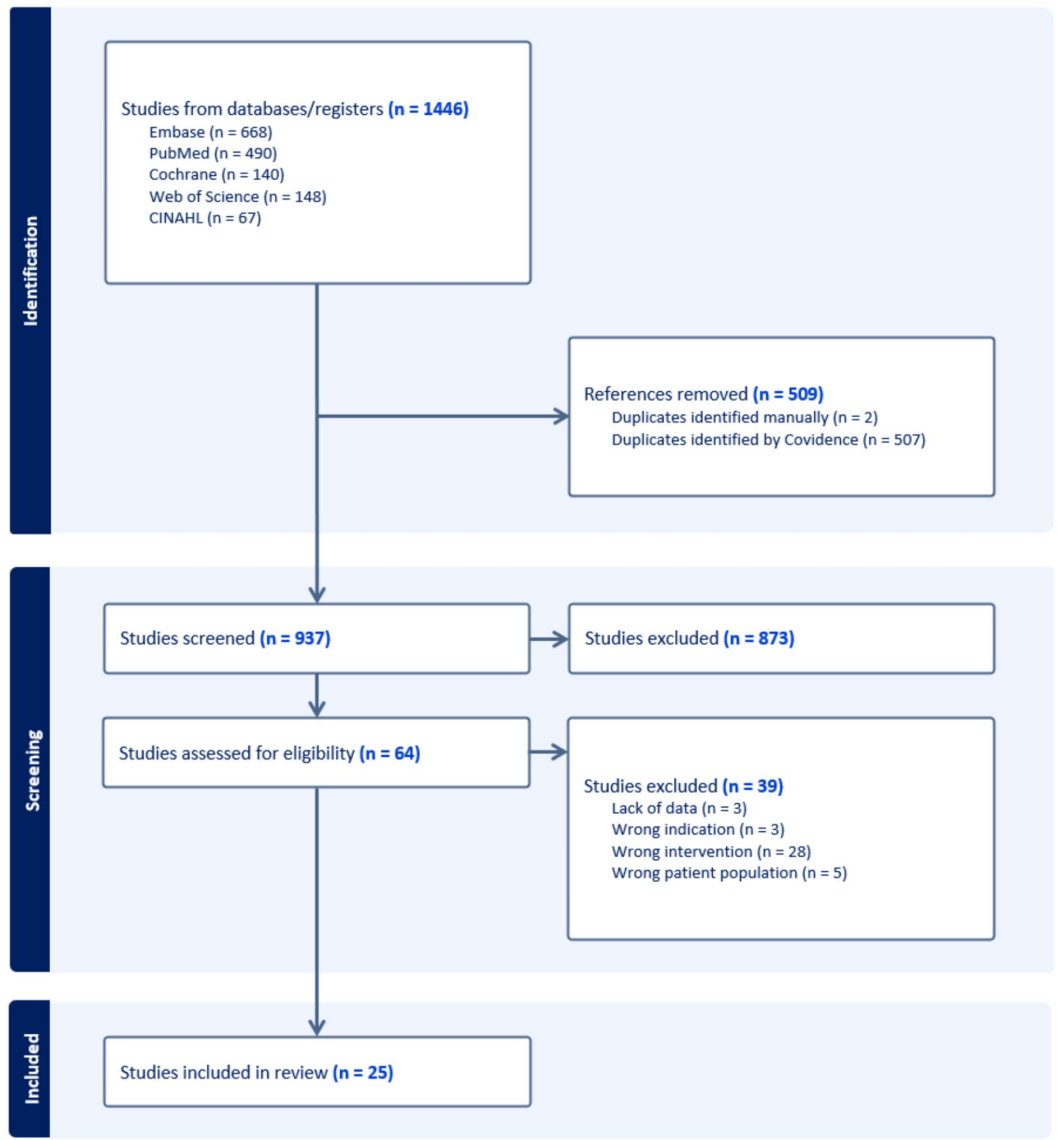
PRISMA flowchart detailing study identification, screening, and inclusion

**Fig. 2:**
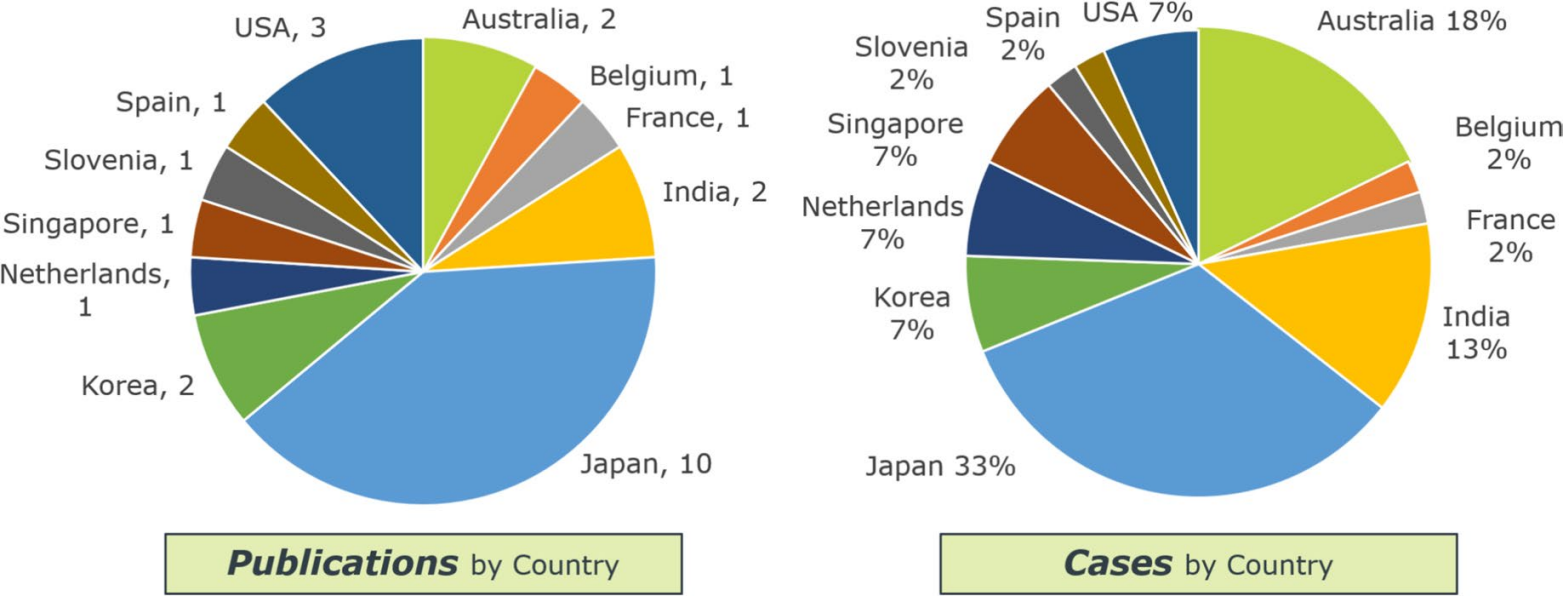
Publications (number) and cases (percentage) by country that met inclusion criteria for the systematic review

The search strategy yielded 1,357 initial entries. After removal of duplicates and ineligible studies, 25 articles fulfilled inclusion criteria, comprising of 45 cases/patients (Fig. 1) [23–46]. Publications spanned 11 countries, with Japan reporting the greatest proportion of cases (Fig. 2).

### Patient characteristics

Patient characteristics and operative metrics are summarised in Table 1 (see Supplementary Table 1 for complete data). The median (interquartile range; IQR) age was 62 (53–70) years and the median (IQR) BMI was 24.2 kg/m^2^ (22–26). Indication for surgery was invasive rectal cancer for 25 cases (55.6%), synchronous malignancy for 18 cases (40.0%) and invasive PC for 2 cases (4.4%). Most cases (33/45; 75.6%) received neoadjuvant treatment, comprising chemoradiotherapy (26/45; 57.8%) or chemotherapy alone (7/45; 20.0%). Specific neoadjuvant chemoradiotherapy regimens were heterogeneously reported. One case of invasive PC had previously received prostate brachytherapy.

**Table 1:** Patient demographics and disease characteristics

Variable	Median (IQR) *(n* = 45)
Age	62 (53–70)
BMI	24.2 (22–26)
Primary Pathology	*n* (percentage)
RC invading prostate	25 (55.6)
Prostate cancer invading rectum	2 (4.4)
Dual pathology	17 (40.0)
Neoadjuvant Therapy	*n* (percentage)
Chemoradiotherapy	26 (57.8)
Chemotherapy	7 (20.0)
Nil/not reported	12 (26.7)

### Surgical approach

Surgical metrics and approaches are summarised in Table 2. Different approaches were reported, such as abdominoperineal resection (APR, 24/45; 53.3%), anterior resection (AR, 12/45; 26.7%) and intersphincteric resection (ISR, 6/45, 13.3%). The rectal resection was performed laparoscopically in 3 cases (6.7%), 2 of which were AR cases and 1 APR case. The colorectal procedure was performed first in the majority of cases (27/45, 60.0% versus 11/45, 24.4%; 11/45 not reported). Considering urinary reconstruction, vesico-urethral anastomosis was performed in most cases (41/45; 86.7%), with variable indwelling urinary catheter duration (range 7–116 days, *n* = 9/45). A minority of APR cases (6/45; 13.3%) underwent bladder neck closure and SPC insertion.

**Table 2:** Surgical metrics and approaches

Variable	Median (IQR) (*n* = 45)
Operative Time (mins)	548 (453–663)
Estimated Blood Loss (mL)	450 (178–661)
Length of stay (days)	15 (10–17)
Rectal resection	*n* (percentage)
APR	24 (53.3%)
AR	12 (26.7%)
ISR	6 (13.3%)
TME	1 (2.2%)
Laparoscopic APR	1 (2.2%)
Laparoscopic AR	1 (2.2%)
Bladder Function	*n* (percentage)
Vesicourethral anastomosis	39 (86.7%)
Bladder neck closure and SPC insertion	6 (13.3%)
First resection	*n* (percentage)
Rectal	27 (60.0%)
Prostate	11 (24.4%)
Not reported	7 (15.6%)

*APR* abdominoperineal resection, *AR* anterior resection, *ISR* intersphincteric resection, *SPC* suprapubic catheter

The median operative time was 548 (360–949) min with a median estimated blood loss of 450 (20–1000) mL. Median length of stay was 15 (4–28) days. Robotic/laparoscopic port placements were variably reported in 37 cases (82.2%) with levels of detail ranging from stating the number of ports used to detailed diagrams. Port placement was typically dependent on the type of rectal resection and resection order.

### Post-operative complications

Post-operative complications were reported for 20 cases (44.4%), the majority of which were minor complications (Clavien–Dindo I–II). Complications are summarised in Table 3. Minor complications occurred in 12 cases (26.7%) and major complications (Clavien–Dindo III–IV) occurred in 8 cases (17.8%). Four cases (8.9%) reported a vesico-urethral anastomotic leak; two cases of APR experienced delayed leaks requiring IDC insertion (Grade IIIa), one APR case was managed with an SPC and 1 AR case was managed with bilateral ureteric catheters. There was 1 case of urine leak without an anastomosis (SPC) requiring bladder neck resuturing (APR). No Grade IV complications occurred.

**Table 3:** Summary of minor and major post-operative complications

Clavien–Dindo classification	Details	Number of cases (*n* = 12)
Minor		
I	Ileus	2
	Minor vesico-urethral anastomotic leakage managed conservatively	1
	Ileus and atrial fibrillation (AF)	1
	AF and hyperglycaemia	1
II	Unspecified	3
	Ileus and surgical site infection	1
	Ileus and lower respiratory tract infection	1
	UTI treated with oral antibiotics	1
	Bleeding peptic ulcer treated with pharmacotherapy	1
Major		
IIIa	Delayed vesico-urethral leak—*Managed with:* IDC insertion SPC insertion Bilateral ureteric catheters	4 2 1 1
	Urinary leak without anastomosis (APR) managed with bladder neck resuturing	1
IIIb	Small bowel loop obstruction (required laparotomy on the 7th post-operative day)	1
	Bowel anastomotic leak (subsequent ileostomy)	1
	Unspecified	1

Considering colorectal complications, there was 1 case of bowel anastomotic leakage requiring covering ileostomy (Grade IIIb). There was 1 case of small bowel obstruction requiring re-exploration on post-operative day 7 (Grade IIIb). One case reported a Grade IIIb complication without specific details of the complication.

### Pathological outcomes

Surgical margin status was well-reported (41/45, 91.1%). Most cases (38/41, 92.7%) reported negative surgical margins (see Table 4). Of those reporting involved surgical margins, 1 case of invasive RC reported a microscopically positive margin at the right postero-lateral side of the rectum. This patient had received historical rectal radiotherapy. Another case of invasive RC reported cauterised rectal tumour cells at the bladder neck margin with clear surgical margins otherwise. A case of synchronous malignancy reported a 1 mm focal positive margin of prostate cancer in the prostate specimen.

**Table 4:** Pathological and oncological outcomes

Variable	*n* (percentage) (total, *n* = 45)
Resection margin status reported	41
R0	38 (92.7)
R1	3 (7.3)
Disease Recurrence Reported	31
Disease free	24 (77.4)
Distant all-cancer recurrence	7 (22.6)
Additional Therapy Reported	12
Adjuvant chemotherapy	8
Salvage chemotherapy	3
Radical cystectomy and ileal conduit	1

*R0* microscopically negative resection margin, *R1* microscopically positive resection margin

### Oncological outcomes

Follow-up duration was reported for 22 cases (46.7%) with a median follow-up of 12 (1–60) months. Thirty-one cases reported on disease recurrence with 24 (77.4%) of these cases disease-free (see Table 4). Of the disease-free cases, 15 cases (62.5%) reported follow-up duration with a median of 12 (6–60) months. Four cases of invasive RC reported disease recurrence (2 cases of lung metastases, 1 case of para-aortic lymph node recurrence, and 1 case of lung and unspecified lymph node recurrence). Two cases of synchronous malignancy reported recurrence of unspecified primary malignancy (1 case of bone metastasis and 1 case of para-aortic and mediastinal lymph node recurrence). A case of synchronous malignancy with a positive prostate margin reported a rising PSA to 0.09 ng/ml after 9 post-operative months.

Twelve cases (26.7%) reported on further post-operative oncological therapies, the majority of which received chemotherapy (3 cases with distant disease recurrence, 7 cases of invasive RC underwent adjuvant chemotherapy, 1 case with synchronous malignancy underwent adjuvant chemotherapy) (see Table 4). One case proceeded to radical cystectomy and ileal conduit with concern for residual bladder-invasive rectal tumour.

### Functional outcomes

Functional outcomes were not reported in most cases (30/45, 66.7%). Urinary function was reported in 14 cases with most reporting continence recovery (11/14; 78.6%). Definitions of urinary continence were variable. Three cases (21.4%) reported urinary incontinence, detailed as requirement of at least one pad per day (*n* = 1), persistence despite pelvic floor exercises (*n* = 1) and stress urinary incontinence without any further detail (*n* = 1). All 3 cases that included erectile function data reported erections sufficient for sexual intercourse in 2 cases and partial erections in 1 case.

## Discussion

This systematic review represents the broadest summary of simultaneous robotic prostatectomy and rectal resection cases. Multi-surgeon familiarity with robotic surgery lends itself to combined operations, particularly in the pelvis, where access for open surgery can be more challenging. Here, we report this combined robotic surgery approach to result in a high rate of negative surgical margins, acceptable rates of mostly minor complications, and promising functional outcomes, reinforcing the potential of this approach in complex pelvic surgery for patients with synchronous or invasive prostate and rectal cancers.

The negative surgical margin rate of 92.7% compares favourably to non-robotic bladder-sparing case series with R0 resection rates ranging from 50 to 95% [9, 10, 12], suggesting that robotic techniques can achieve oncological outcomes equivalent to, or even better than, those of traditional open surgeries. The disease-free rate of 77.4% at follow-up underscores the oncological efficacy of simultaneous resection in a population of high oncological risk. This is notably greater than Turner and colleagues’ open simultaneous resection disease-free rate of 36.4% [12], although data and follow-up duration of the robotic data are limited. Previous case studies in open pelvic surgery showed no survival difference between bladder-preserving dual resection and total exenteration [9]. We are optimistic about the long-term survival of these cases, particularly since there has been significant improvement in survival following pelvic exenteration surgery over the past 30 years despite increasing case complexity [47].

An overall perioperative complication rate of 44.4% compares favourably to open dual resections (73%) [12]. The major complication rate of 17.4% is similar when compared to robotic multi-visceral resections for RC, regardless of bladder-sparing. Shin and colleagues reported a major complication rate of 14% [48] and Crolla and colleagues reported a major complication rate of 21.7% [24]. One (5%) bowel anastomotic leakage was reported in this series amongst 20 bowel anastomoses. Despite the heterogeneity of complication reporting, this seems comparable to multi-organ RC resection data reported by Crolla and colleagues, whereby 10% of bowel anastomoses suffered leakage [24].

More than half (57.8%) of the simultaneous resections were performed in an irradiated pelvis which is comparable to non-robotic series reporting neoadjuvant radiotherapy rates ranging from 63.6 to 77.1% [9, 12].

While a urinary anastomotic leakage rate of 8.9% (considering pre-operative radiotherapy use of at least 57%) is comparable to contemporary non-robotic cases (9.1%) [12], this is significantly improved from historical literature citing anastomotic leakage rates as high as 50% [10]. Anastomotic leakage rates in salvage radical prostatectomy alone after radiation therapy range from 4% [49] to 12.4% [50], suggesting that multi-organ resections may not alter anastomotic leakage risk. In general, these findings suggest that robotic assistance may reduce the risk of complications, especially in technically demanding urinary or bowel anastomoses within an irradiated pelvis. This emerging technique is expected to improve further with increased adoption of this technique, likely with reduced complications.

Considering short–medium-term functional outcomes, despite limited reporting, the available data indicate that urinary continence was achieved in 78.6% of cases, a substantial improvement over Turner and colleagues who reported a continence rate of 36.3% in an 11-case series of open procedures [12]. Impotence rates of 45% have been cited amongst open simultaneous resections [12]. While the improved precision and capability of the robotic approach may improve nerve preservation [51], current functional data were limited (reported in only 33.3% of cases), likely reflecting a gap due to the emphasis on surgical technique and short-term oncological outcomes of these novel procedures. This relative absence of data in this cohort means that patient counselling is challenging; however, despite the positive available data (3 cases, all had erectile recovery), outcomes after salvage prostatectomy would indicate low-no erectile recovery should be advised until further high quality data become available.

Quality of life (QoL) assessment in future case series may supplement understanding of functional outcomes with bladder preservation. Wiig and colleagues reported post-operative IPSS QoL index after open simultaneous resections [10], with only 1 patient reporting ‘mixed’ (other cases either ‘delighted’ or ‘pleased’). Patients wanting bladder preservation may be willing to accept a greater compromise in urinary function to avoid urinary diversion so patient counselling on the options is of utmost importance. However, these procedures are generally performed for patients with a favourable life expectancy so medium- (3–5 years) and long- (10 + years) term oncological and functional outcomes require further characterisation in line with other high quality prostate cancer trials [52]. In particular, improvements [53] and decline (after salvage radiotherapy [54]) in medium term outcomes have been noted in some surgical studies and would be valuable to characterise for this cohort.

The strengths of this review include wide-ranging analysis of available cases across multiple countries using a systematic approach to data extraction and analysis. However, limitations include heterogeneity in reporting and the small number of cases available in the literature. This variability in data quality and the absence of standardised outcome measures restricts the ability to perform detailed meta-analyses and limit the generalisability of the findings of follow-up (reported in 46.7% of cases; median duration 12 months) and key clinical outcomes, such as functional outcomes (reported in 33.3% of cases). The quality of available studies was low and high heterogeneity limited quantitative synthesis by meta-analysis; therefore, future studies should be of a similar standard to other prostate cancer studies to facilitate more robust meta-analyses and help establish clearer guidelines for practice. The small sample size of reported cases restricts the statistical power of our findings and highlights the need for multi-centre registries to build a more comprehensive data set. While the novelty and high expertise required for this procedure limits large sample sizes, we encourage formation of high quality multi-centre registries to better capture clinically relevant data in larger cohorts to enable meaningful outcome reporting and patient-centred shared decision making, similar to the SATURN registry for male incontinence surgery [55].

In conclusion, simultaneous robotic-assisted prostatectomy and rectal resection is a promising surgical option for either synchronous or invasive RC and PC, evidenced by the negative surgical margin rate and low complication rates. While this systematic review provides a foundation for the use of robotic-assisted techniques in simultaneous resection, more standardised reporting and longer follow-up are needed to fully ascertain the long-term benefits and refine patient selection criteria. Future studies should aim to address these gaps through prospective data collection and the development of registries that can track long-term oncological and functional outcomes.

## Supplementary Information

Below is the link to the electronic supplementary material.

Supplementary file1 (XLSX 16 KB)

## Data Availability

No data sets were generated or analysed during the current study.
